# Default mode network and frontolimbic gray matter abnormalities in patients with borderline personality disorder: A voxel-based meta-analysis

**DOI:** 10.1038/srep34247

**Published:** 2016-10-03

**Authors:** Xun Yang, Liyuan Hu, Jianguang Zeng, Ying Tan, Bochao Cheng

**Affiliations:** 1 School of Sociality and Psychology, Southwest University for Nationalities, Chengdu, China; 2 Huaxi MR Research Center (HMRRC), Department of Radiology, West China Hospital of Sichuan University, Chengdu, China; 3 School of Accounting, Southwestern University of Finance and Economics, Chengdu, China; 4 School of Computer Science and Technology, Southwest University for Nationalities, Chengdu, China; 5 Department of Radiology, West China Second University Hospital, Sichuan University, Chengdu, China

## Abstract

Specific frontolimbic abnormalities are hypothesized to underlie the etiology of borderline personality disorder (BPD). However, findings from neuroimaging studies were inconsistent. In the current study, we aimed to provide a complete overview of cerebral microstructural alterations in gray matter (GM) of BPD patients. A total of 11 studies were enrolled, comprising 275 BPD patients and 290 healthy controls (HCs). A meta-analysis was conduct to quantitatively estimate regional GM abnormalities in BPD patients using the seed-based *d* mapping (SDM). Meta-regression was also conducted. Compared with HCs, the BPD patients exhibited increased GM mainly in bilateral supplementary motor area extending to right posterior cingulated cortex (PCC) and bilateral primary motor cortex, right middle frontal gyrus (MFG), and the bilateral precuneus extending to bilateral PCC. Decreased GM was identified in bilateral middle temporal gyri, right inferior frontal gyrus extending to right insular, left hippocampus and left superior frontal gyrus extending to left medial orbitofrontal cortex. The mean age of BPD patients were found nagativly associated with GM alterations in right MFG. Our findings suggested that BPD patients have significantly GM abnormalities in the default mode network and frontolimbic circuit. Our results provided further evidences in elucidating the underline neural mechanisms of BPD.

Borderline personality disorder (BPD) is a common axis II psychiatric disorder, which is characterized by instability of mood, social relationships, self-image and impulsivity^1^. Other symptoms usually include intense fear of abandonment, sense of emptiness, complex dissociation, aggressive, intense anger and irritability. The incidence of BPD is approximately 1% in the general population^2^ and the prevalence of BPD in psychiatric settings is higher (upward of 25%)^3^. Moreover, a highly prevalent rate of comorbidity with other mental disorders have been frequently reported in BPD patients such as alcohol abuse (AD), posttraumatic stress disorder (PTSD), obsessive-compressive disorder (OCD), major depression (MD) and substance use disorders, which affects a person’s general health and lead to increased social and economic burdens^4^.

Previous BPD studies mainly focus on the psychological aspects of the disorder from trait to triggers and risk factors. Until recently, there have been a growing interests in identifying the neurobiological mechanisms with neuroimging tools. Due to the instability characters of BPD, structural MRI studies are potentially more amenable to determine the common neuroanatomical changes underpinning the BPD symptoms. Especially, the study of gray matter (GM) could indicate the amount of regional GM which is recognized as reliable measure to reflect the abnormal cerebral microstructure^5^ and hold promise as endophenotypes for specific personality disorders.

Several strucutal MRI studies have revealed the GM reduction in the frontal cortices including orbitofrontal cortex^6^,^7^,^8^, the anterior cingulate cortex^8^, and the parietal cortex^9^. With regard to subcortical limbic structures, GM reduction in amygdalar^10^,^11^ and hippocampus^10^,^12^ have also been reported in patients with BPD. Both human and experimental animal findings suggested that the amygdala is central to the generation and maintenance of negative emotional responses^13^, and the frontal deficits could lead to increased difficulty in controlling negative emotions (down-modulation)^14^,^15^. Therefore, these results suggested that BPD might result from structural abnormalities in the frontolimbic circuit and subsequent impairment of emotional regulation^16^.

Although group studies have reported widespread brain structural abnormalities of BPD paitents in frontolimbic areas, the results are heterogeous. For example, some studies fail to found GM alternation in amygdalar^17^, while others found reduced GM in amygdalar^7^,^18^,^19^. These divergences are most likely due to the sample differences such as small sample sizes, comorbidities, age and gender. On the other hand, Vollm and colleagues (2009) reported that men with BPD had reduced GM in the frontal, temporal and parietal cortices^20^, while BPD female were reported to have no significant GM alternation in frontal cortices in a voxel-based morphometry study^18^. Thus, these results must be understanding with caution regarding the effect of gender.

Voxel-based Meta-analysis can integrate multiple original morphology studies of one type and examine the associate between important demographic and clinical varibles and cerebral microstructual changes. Two prior studies^21^,^22^ evaluated the morphological changes of amygdalar and/or hippocampal in BPD across the largest group of adult studies, using the traditional meta-analytic methods. This traditional meta-analysis can provide the analysis of magnitude, and reflect the average individual effect size across the sample of studies included in the synthesis^23^, but this analysis decide priori regions to investigate (e.g. amygdala and/or hippocampus) which lead to a loss of valuable information. A recently developed meta-analytic method called signed differential mapping (SDM) can provide unbiased assessment of cerebral structural differences on a point-by-point basis over the whole brain^24^.

As the research stands, it is evident that more concrete findings are needed in order to gain a firmer understanding of the neurobiology mechanism underpin BPD. Thus, our present study quantitatively review the published voxel-based morphometry (VBM) studies on BPD to identify the consistent cerebral regional GM abnormalities using the approach of effect size SDM (ES-SDM). Furthermore, by combining with meta-regression methods, we expect to characterize the impact of demographic and clinical variables on brain microstructure alternation.

## Results

### Characteristics of the included studies and samples

**Figure 1:**
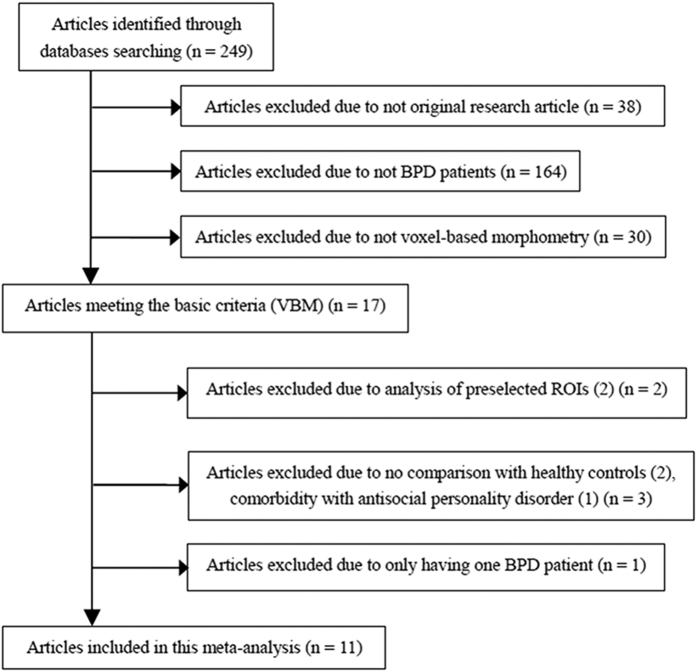
Flow diagram of inclusion and exclusion process of selected articles of VBM studies in patients with BPD. *Abbreviation*: VBM, voxel-based morphometry; BPD, borderline personality disorder.

As shown in Fig. 1, our search strategy identified 249 studies. Including which, 11 studies comparing whole-brain differences of 275 BPD patients and 290 healthy controls (HCs) met the inclusion criteria were included in our study^6^,^8^,^12^,^16^,^18^,^25^,^26^,^27^,^28^,^29^,^30^. The mean age of BPD patients (29.11 ± 5.34 years) and of HCs (28.62 ± 5.79 years) had no satistical difference (*p* = 0.836). The percentage of female BPD patients (226 patients, 82.18%) and HCs (234 control subjects, 80.68%) were no statistical difference (X^2^ = 10.333, *p* = 0.412). Detailed demographic and clinical characteristics of all included studies has been clearly presented in Table 1.

### Regional alternation of grey matter

**Figure 2:**
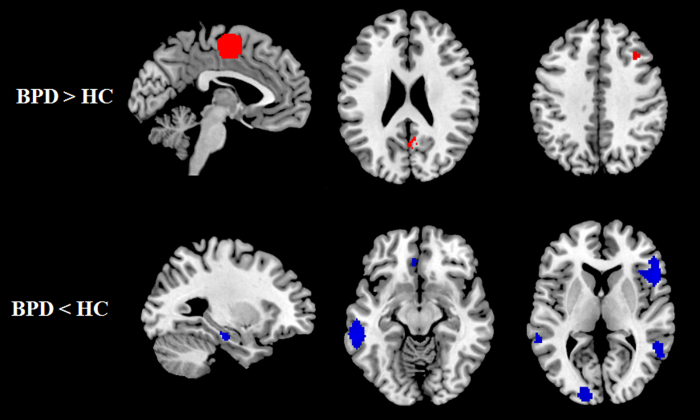
Regions showing gray matter alteration in BPD patients compared with health controls. Areas with increased gray matter relative to controls are displayed in red, and areas with decreased gray matter are displayed in blue. *Abbreviation*: BPD, borderline personality disorder; HC, healthy control.

Relative to HCs, the BPD patients exhibited increased GM in the bilateral supplementary motor area (SMA) (extend to right posterior cingulated cortex, (PCC) and bilateral primary motor cortex, PMC), right middle frontal gyrus (MFG), and the bilateral precuneus (PrCC) (extend to bilateral PCC), and decreased GM were identified in the bilateral middle temporal gyri (MTG), the right inferior frontal gyrus (IFG) (extend to right insula), the left hippocampus (HIP), the left middle occipital gyrus (MOG) and the left superior frontal gyrus (SFG) (extending to left medial orbitofrontal cortex, OFC) (Table 2, Fig. 2). As shown in Table 3, both the subgroup analyses and the systematic whole-brain jack-knife sensitivity analysis of the pooled studies presented that the GM reduction in the left MTG, right IFG, and GM enlargement in the right SMA were highly replicable, being observed in all the inclusion stuides. Those results in the right MTG, left HIP and the right precuneus remain significantly in all but one combination of data sets. Left SFG and right MFG remain significant in all but two one combinations of data sets.

### Analysis of heterogeneity and publication bias

A number of regions showed divergence in heterogeneity analysis such as right insula, right supplementary motor area, right middle temporal gyrus, and left superior frontal gyrus. Analysis of publication bias revealed that the Egger test was not significant for the right insula (p = 0.937), right supplementary motor area (p = 0.719), right middle temporal gyrus (p = 0.661), and left superior frontal gyrus (p = 0.155).

### Subgroup meta-analysis

Considering the methodological variants (e.g. GM volume/density analysis, different smooth kernel), subgroup analysis were conducted several times. The abovementioned results remained largely unchanged when the analyses were repeated and limited to methodologically homogenous groups of studies (Table 4). Two additional significant cluster in the right putamen (Montreal Neurological Institute [MNI] coordinates x = 30, y = −10, z = 2, SDM Z = −1.063, p = 0.00006), and left superior occipital gyrus (x = −14, y = −94, z = 10, SDM Z = −2.084, p = 0.0004) emerged in the subgroup analysis of studies that using DARTEL algorithm and of studies that with modulation step.

In seven datasets recruite medication-naive BPD patients that including 163 patients without medication and 164 HCs. Relative to HCs, the subgroup analysis revealed that medication-naïve BPD patients showed increased GM in right SMA (extend to right PCC), and the left precuneus, and decreased GM in the left MTG, and the right IFG (See Table 4).

Whole brain jackknife sensitivity analysis revealed these results were highly replicable, and these finding were preserved in all seven combinations of data sets, expect left precuneus and left MTG remain significant in all seven but one combinations of data sets.

### Meta-regression

**Figure 3:**
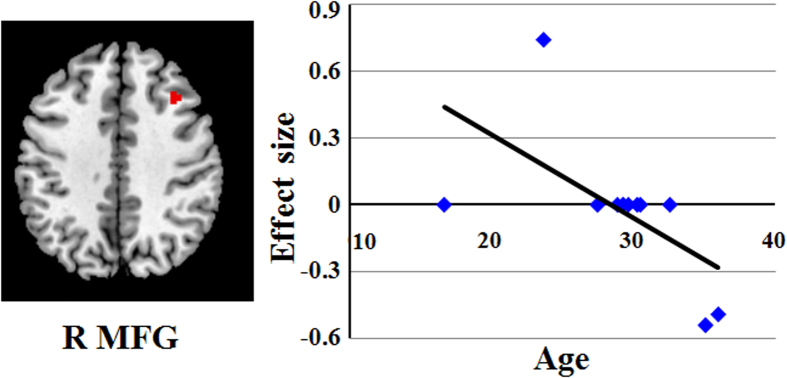
Results of the meta-regression analysis showing a significant inverse association between age and grey matter in the right middle frontal gyrus. *Abbreviation*:MFG, middle frontal gyrus.

Using meta-regression analysis, we investigated the effects of age and gender on cerebral GM alternation in BPD patients, which were available for all data sets. The mean age of BPD patients was significantly negatively associated with GM alterations in right MFG (r = −0.607, permutation-derived p = 0.0002; Fig. 3). The gender of BPD patients was not linearly associated with GM alternation.

Some clinical variables could not be studied in the meta-regression analysis because data sets were not available^31^. We also failed to reveal whether BPD symptom severity was associated with the reported GM alternation because of a wide range of different measures used by inclusion studies.

## Discussion

In our current study, quantitative SDM meta-analytic methods were used to synthesize findings from 11 pooled VBM studies for a meta-analysis of GM abnormalities in BPD patients. The study enrolled sufficient number and up todate high-quality studies. Relative to HCs, BPD patients exhibited GM reductions within the region comprising the bilateral MTG, the right IFG, right insula, left hippocampus, left middle occipital gyrus, the left SFG and left medial OFC. Increased GM were identified in the bilateral SMA (extend to right PCC and bilateral PMC), right MFG and the bilateral PrCC (extend to bilateral PCC). Subgroup analysis revealed that the medication-naive BPD patients have less GM alterations than overall BPD patients. In addition, the mean age of BPD patients was significantly negative associated with GM alterations in right MFG.

The most prominent of our findings is that the enlarged GM were found in the bilateral PCC and the PrCC (two key nodes of default mode network, DMN) of BPD patients with or without medication. DMN has been observed displaying greatest levels of activity when at rest, hypoactivity during task-based stimulation and thought to be involved in self-referential processing, inner speech, emotional control and episodic memory^32^. Recent functional MRI studies have found aberrant activity and functional connectivity of DMN in BPD patients during emotion or self-related tasks^33^,^34^,^35^. Particularly, negative emotional stimuli could evoke stronger activity in the PCC and frontal cortex, as well as decreased activity was found in the amygdala of BPD patients^36^,^37^. And increased functional connectivity was also found in BPD patients between the precuneus and the left inferior frontal lobe, left precentral/middle frontal, and left middle occipital lobes during resting-state^35^. The DMN chaos could help to understand the link between neurobiological disturbances and clinical symptoms in BPD patients.

As the hub of the DMN and the limbic system, PCC play an key role in assessing the emotional salience and self-relevance of external stimulus, memory encoding and its interactions with emotions^38^. Functional MRI study has revealed that emotional stimuli could activate PCC^39^. Increased activity and metabolism in PCC make this area more vulnerable to illnesses such as Alzheimer’s disease, Parkinson’s diseases, schizophrenia and other mental disorders^40^.

As another crucial node in the DMN, PrCC has been suggested to involve in reflective, self-related processing, empathy, awareness and conscious information processing^41^. Considering the previous report of increased connectivity between the precuneus and the frontal cortex^35^, the enlargement GM in PrCC of our results may have implications in terms of extensive processing of internal thoughts and self-referential information in BPD.

PrCC has also involved in intentional, self processing^42^. The essential function of self reflection for PCC/PrCC is compatible with a pivotal role for this region in conscious awareness. Indeed, self reflection may be the primary substrate for conscious awareness as located in the PCC/PrCC. Thus, the disturbances of PCC and PrCC maybe pivotal for self-focus related conscious information processing, which could underlie core symptom of BPD, such as unstable of self image.

In addition to DMN structures, we found enlarged GM in the bilateral SMA and bilateral PMC. PMC works in association with other motor areas such as SMA. And these two regions are involved in planning complex movements and in coordinating movements involving both hands. Previous study also found SMA GM increased in BPD patients^43^. However, the detail function of SMA and PMC in BPD remains largely unclear.

In our current voxel-based meta-analysis, BPD patients exhibited reduced GM mainly in the frontolimbic circuit (the right IFG, right insular, left hippocampus, left middle occipital gyrus and the left SFG and left medial OFC) and temporal cortex (bilateral MTG). GM deficits in prefrontal areas of our current study are in accordance with several previous studies^6^,^16^,^26^,^28^. Some of these studies found additional GM deficts extending from the frontal cortices to the limbic system^16^,^28^. Previous studies had linked impulsivity and emotional instability features of BPD to the chaos of integrated frontolimbic functions^44^ and suggested that emotional dysregulation is caused by prefrontal deficits, hyperactivity of the limbic system, or a combination of both^45^. In particular, frontolimbic areas are implicated in affective regulations.

The abnormalities in the posterior parietal areas which have been linked to the presence of dissociative symptoms^46^. And evidence of reduced temporal cortices volumes have also been found in impulsive-aggressive offenders^10^ and also in BPD males^29^.

As a component of the limbic system, the insula cortex is engaged in various emotional and cognitive functions^47^. Soloff *et al.* reported BPD patients had significantly diminished GM in insula cortex^48^. Takahashi *et al.* reported insula cortex volume does not significantly differ in early BPD, but might have a relationship with violent and impulsive behavior^49^. These evidence implicated that insula cortex might involve in the psychopathology of BPD.

As another key node of limbic system, hippocampus was reported loss nearly 11% of size in a recent ROI-based meta-analysis of BPD^21^. Our findings revealed a smaller left hippocampus but not right hippocampus of BPD patients. It is possible that a greater reduction in left hippocampus could be a biological feature specific to BPD. As the hippocampus and other medial temporal cortex involving regulation of stress-associated neural systems, volumetric reductions in these areas may result in trauma related neuro-cognitive deficits and other symptoms of BPD. In particular, frontolimbic areas are implicated in affective regulations and abnormalities in posterior parietal areas have been suggested as a possible correlated of affective and dissociative symptoms^46^. Taken together, these evideces suggested that dysfunctional frontolimbic brain regions underlie the “emotional turmoil” in BPD patients.

Both the pooled overall BPD and the medication-naive subgroup exhibited increased GM in the right SMA (extend to PCC) and the left PrCC, and exhibited decreased GM in the left MTG, and the right IFG. Other regions such as right MFG. right MTG, left MOG, and hippocampus fail to find GM alternation in medication-naive BPD subgroup. BPD patients have diverse symptoms due to a high rate of comorbidities with other psychiatric disorders and differed treatment strategies. Especially, medications with anti-cholinergic and sedative side-effects would impair cognitive function^50^. In order to figure out the medication efffects in BDP patients, we conduct the subgroup meta-analysis. Our findings suggest that psychotropic medication has a substantial impact on GM alteration in BPD patients.

A Behavior study has revealed that the bordline symptoms and suicidality/self-injury maybe preceded with age^51^. Our fingdings that the mean age of BPD patients negatively associate with GM in the left MFG are consistent with clinical impressions regarding the course of BPD symptoms. These results also indicate that the old adult BPD individuals tend to have smaller GM in MFG.

Contrary to our expectations, this meta-analysis did not reveal significant GM alternation of BPD in some frequently mentioned regions such as amygdala and ACC, which has been consistently implicated in previous functional neuroimaging studies of BPD and constitutes the basis of the most widely accepted neurobiological model of BPD. Accumulating evidences have indicated that BPD exhibited reduced amygdalar volumes, and exaggerated amygdalar response, as well as reduced prefrontal/ACC inhibition to social and emotional stimuli^12^,^26^. However, the literatures povided heterogeneous results on both structures. For example, despite of significant volume reduction of the amygdala were revealed^18^,^19^,^26^, structural increases have also been reported^26^. In our present study, only 3 of the 11 included dataset identified structural changes in amygdala^8^,^18^,^26^ and ACC^12^,^26^,^29^. There are several plausible explanations for this divergence, which are not necessarily mutually exclusive, for the lack of robust structural changes in these two structures. First, this inconsistency may partly be due to heterogeneity of demographic characteristics (such as age, and sex). One study reported only male, but not female, BPD subjects have decreased gray matter concentrations in ACC bilaterally^8^, which may be related to the neurobiology of impulsivity and aggression. In addition, age effects on brain areas resulting from differential developmental stage may contribute to heterogeneity in morphometric changes in BPD because that reduced ACC volume have been reported in adolescent patients with a first presentation of BPD, but not in adult patients^52^. Examining adult samples has the potential to increase the confounding influences of prolonged illness, treatment, and recurrent or chronic comorbidities. Third, ACC structure has been found to vary in location and complexity across individuals^53^, thus the high interindividual variation of the ACC makes spatial normalization in VBM more difficult and susceptible to errors, which may explain inconsistencies results for ACC volume in patients with BPD^12^. Forth, voxel-wise meta-analytical methods could provide excellent control for false positive results, however, it is more difficult to avoid false negative results. Finally, as in any other coordinate-based method, the number of secondary peaks included would affect the accuracy of results”^24^.

Although the GM alterations in BPD with or without comorbid with Axis II disorders were clearly demonstrated in our meta-analysis and subgroup analysis, structural MR studies of BPD may be confounded by Axis I comorbidities. As BPD is considered as a heterogeneously disorder^24^ and is associated with high comorbidity rates. While the most common seen comorbidities with BPD samples were mood disorder, anxiety, eating disorder, posttraumatic stress disorder and substance use disorders in Axis I, and cluster C personality disorders in Axis II^54^,^55^. The included datasets in our current meta-analysis mainly presented with Axis I comorbidities, and only few samples reported comorbidity with Axis II diagnoses. Given the high prevalence of BPD comorbidity rates of pooled Axis I disorders, it was hardly to figure out this impact. Further longitudinal studies examine the association between brain morphological changes and the long term course and comorbid outcome of BPD will are needed.

Finally, the included samples varied in terms of the disease duration and onset of illness. We hardly perform a separate sub-group or meta-regression analysis to investigate these variables in this study due to lacking sufficient available information^31^. A larger sample size will give us the opportunity to stratify for these variables and check their impact is warranted.

## Conclusion

In the current meta-analysis, we revealed GM alternation in BPD patients relative to HCs. Our results suggested that patients with BPD have significantly GM abnormalities in the DMN and frontolimbic structures. The GM abnormalities observed in our BPD samples underpin psychological functions that are compromised in BPD patients. Considering the structural abnormalities often underline the function chaos, we suppose that the malfunction of both DMN and frontolimbic network might implicated in etiology of BPD.

## Methods

### Study selection

Using PubMed (http://www.pubmed.org), Google Scholar (http://scholar.google.com), Embase (https://www.embase.com), and Science Direct (http://www.sciencedirect.com), we conducted comprehensive literature search for published MRI studies of BPD in English between January 2000 and November 2015. Key words used the following strategies: 1) borderline personality disorder (BPD); 2) voxel-based morphometry, or VBM, or morphometry, or volumetry, or grey matter, or structural MRI. In addition, the references cited in the selected articles were also manually checked for any possible inclusions.

Studies were selected for meta-analysis employing the following inclusion criteria: (a) utilize whole-brain voxel-based morphometry (VBM) to analyze the GM alterations; (b) comparison of BPD patients with HCs; and (c) The results reported the Talairach or Montreal Neurological Institute (MNI) coordinates of the activation regions. Studies were excluded from our study as follows: (a) theoretical articles, literature reviews or any other meta-analysis, and studies that re-analyzed previously published data; (b) non-English or unavailable full-text studies. Observational Studies in Epidemiology (MOOSE) guidelines for meta-analyses were followed in this study^56^.

### Meta-analysis of the studies

Voxel-wise meta-analysis was performed on the selected studies to compare the GM alternation between BPD patients and HCs using seed-based *d* mapping (SDM, version 4.22 for meta-analysis) (http://www.sdmproject.com/software). As an effectively meta-analytic method, SDM has been widely used in a number of MRI meta-analysis studies^57^,^58^,^59^. This software could well combine peak coordinates and statistical parametric maps and use standard effect size and variance-based meta-analytic calculations. The detail meta-analysis preocess had been clearly clarified in our newly published meta-analysis article^60^, and which included several steps as follows: First, the same threshold across the whole brain was set^31^; Second, SDM table containing the coordinate information was created; Third, Monte Carlo brain maps were generated; Finally, other related analyses including heterogeneity analysis, subgroup analysis, jackknife sensitivity analysis, and meta-regression were performed.

The heterogeneity analysis of each result was examined using a random effects model with Q statistic (X^2^) distribution converted to z values and tested with a permutation approach. We examined the possibility of publication bias for brain regions showing GM alteration using the Egger test^61^.

The meta-analysis repeated several times including only methodologically homogenous studies in order to control any possibly methodological differences between included studies. In particular, these analyses repeated for the studies which using 1.5T MR scanner, for which acquired the images with a slice thickness of 1.0 mm, for those using DARTEL algorithm, for which using a 8 mm smoothing kernel, for which performing an additional modulation step (i.e. inference of absolute GM volume instead of GM density), and for which reporting coordinates corrected for multiple comparisons.

A systemic whole-brain voxel-based jackknife sensitivity analysis was conducted to test the replicability of results in different studies. The analysis was repeated 20 times, with a different study being excluded each time. If the findings of previous SDM significant results could be replicated in all or most included studies, the findings might be very replicable and conclusive.

We also conduct a sub-group analysis of medication-naive BPD patients. Recruiting such individuals strategically help to reduce previous antidepressant treatment as confounder that may dilute the neuropathological findings in BPD.

Meta-regression analyses were examined to test the potential effects of the demographic and clinical variables on the cerebral GM alternations (i.e., the mean age of patients and controls, gender ratios of both groups, scores of Barratt Impulsiveness Scale), which was weighted by the square-root of the sample size and restricted to predict only possible SDM values (i.e., from –1 to 1). In order to avoid spurious results^31^, we cut down the probability threshold to 0.0005 and required abnormalities to be detected both in the slope and in one of the extremes of the regressor, then discarded findings in regions other than those detected in the main analyses^31^. Finally, regression plots were visually inspected to discard fittings driven by too few studies.

## Additional Information

**How to cite this article**: Yang, X. *et al.* Default mode network and frontolimbic gray matter abnormalities in patients with borderline personality disorder: A voxel-based meta-analysis. *Sci. Rep.* **6**, 34247; doi: 10.1038/srep34247 (2016).

## Figures and Tables

**Table 1:** Demographic and clinical characteristics of participants in the 11 VBM data sets included in the meta-analysis.

Study	Patients with BPD				Healthy controls	
	No. (% female)	Mean age, yr	Medication	Comorbidity	No. (% female)	Mean age, yr
Bertsch *et al.*	13 (0)	28.9	N	Positive (APD; SA; PTSD)	14 (0)	26.1
Brunner *et al.*	20 (100)	16.7	Y	Positive (AD^2^; CD; ED; MD; SA)	20 (100)	16.8
Kuhlmann *et al.*	30 (100)	23.7	N	Positive (AD^1^; AD^2^; APD^1^; APD^2^; CPD; DD^1^; DPD;ED; HPD; NPD; PPD; PTSD; RSA)	33 (100)	24.4
Labudda *et al.*	33 (100)	30.51	Y	Positive (APD^2^; BN; DPD; GAD; OCD; OCPD;PD; PPD; PSA; SP^1^; SP^2^; SD^1^)	33 (100)	31.21
Minzenberg *et al.*	12 (41.7)	30.3	N	Positive (APD^1^; APD^2^; HPD; IED; NPD; OCD; PD; PPD; PTSD; SPD)	12 (50.0)	30.7
Niedtfeld *et al.*	60 (100)	29.67	N	Positive (PTSD)	60 (100)	28.5
O’Neill *et al.*	20 (100)	32.6	Y	Positive (MDD)	21 (100)	30.1
Rossi *et al.*	26 (61.5)	36	Y	Positive (AA; APD^1^; DD^1^; DPD; ED; OCD; SA)	40 (52.5)	40
Rüsch *et al.*	20 (100)	29.3	N	Positive (AD^2^; DD^2^; ED; MDD; PTSD; SD^2^)	21 (100)	28.4
Soloff *et al.*	34 (64.7)	27.5	N	Positive (AUD; DD^2^; MDD; PTSD)	30 (63.3)	25.6
Völlm *et al.*	7 (0.00)	35.1	N	Positive (other personality disorder)	6 (0.00)	33

*Abbreviation*:AA, alcohol abuse; AD^1^, adjustment disorder; AD^2^, anxiety disorder; APD^1^, antisocial personality disorder; APD^2^, avoidant personality disorder; AUD, alcohol use disorder; BN, bulimia nervosa; BPD, borderline personality disorder; CD, conduct disorder; CPD, compulsive personality disorder; DD^1^, depression disorder; DD^2^, dysthymic disorder; DPD, dependent personality disorder; ED, eating disorder; GAD, generalized anxiety disorder; HPD, histrionic personality disorder; IED, intermittent explosive disorder; MD, mood disorder; MDD, major depression disorder; N, all patients were without psychotropic drugs; NPD, narcissistic personality disorder; OCD, obsessive-compulsive disorder; OCPD, obsessive-compulsive personality disorder; PD, panic disorder; PPD, paranoid personality disorder; PSA, past substance abuse; PTSD, posttraumatic stress disorder; RSA, remitted substance disorder; SA, substance abuse; SD^1^, somatization disorder; SD^2^, substance dependence; SP^1^, social phobia; SP^2^, specific phobia; SPD, schiaotypal personality disorder; Y, not all patients were without psychotropic drugs.

**Table 2:** Regional differences in grey matter between patients with BPD and healthy controls in the meta-analysis.

Brain regions*	Maximum			Cluster	
	MNI coordinates x, y, z	SDM value	p value	Number of voxels	Breakdown
BPD > control					
R SMA & R PCA	2, 4, 60	1.651	0.00003	1161	R SMA, BA 6 L SMA, BA 6; CC R SFG, dorsolateral, BA 6 R PCA, BA23,24 L PMC, BA 4 R PMC, BA4
R dorsolateral MFG	36,22,40	1.40	0.002	96	R dorsolateral MFG, BA 46
B precuneus	2, −52, 34	1.078	0.0035	84	L precuneus, BA 7; R precuneus, BA 23 R median network, cingulum L PCA; R PCA, BA23
BPD < control					
R IFG, R insular	52, 22, 6	−2.129	0.00066	602	R IFG, opercular part, BA 45, R IFG, triangular part, BA 48,44,47 R insula, BA47
L MTG	−60, −38, −6	−2.289	0.0002	579	L MTG, BA21,20,22 L ITG, BA20
R MTG	54, −56, 12	−2.161	0.00055	334	R MTG, BA 21,22,37 R STG, BA 22, 42
L MOG	−14, −92, 12	−1.983	0.0014	283	L MOG, BA17,18
L hippocampus	−26, −20, −18	−2.115	0.0007	55	L hippocampus, BA20 L median network, cingulum
L SFG, medial OFC	−4, 34, −12	−1.774	0.0042	16	L SFG, medial OFC, BA 11

*Abbreviation*: SMA, supplementary motor area; CC, corpus callosum; PCA, posterior cingulate cortex; PMC, primary moter corex; MFG, middle frontal gyrus; IFG, inferior frontal gyrus; MTG, middle temporal gyrus; ITG, inferior temporal gyrus; STG, superior temporal gyrus; MOG, middle occipital gyrus; SFG, superior frontal gyrus; OFC, orbitolfrontal cortex; BA, Brodmann area; BPD, borderline personality disorder; L, left; R, right; SDM, seed-based *d* mapping; MNI, Montreal Neurological Institute.

^*^Regions identified by meta-analysis of coordinates from 11 data sets (voxel-wise p < 0.005 and voxel size > 10).

**Table 3:** Subgroup analyses and Sensitivity analyses.

	Decreased gray matter					Increased gray matter	
	L MTG	L MTG	R IFG	L hippocampus	L SFG	R SMA	R MFG
Subgroup analysis							
1.5 T MR scanner (n = 5)	Y	Y	Y	Y	N	Y	Y
slice thickness at 1.0 mm acquisition (n = 6)	N	Y	Y	N	Y	Y	Y
DARTEL algorithm (n = 5)	Y	N	Y	N	Y	N	Y
8 mm smoothing kernel (n = 5)	N	Y	Y	N	Y	N	N
uncorrection for multiple comparison (n = 7)	Y	Y	N	N	Y	Y	Y
Studies with modulation step (n = 9)	Y	N	Y	Y	N	N	Y
Studies only comorbid with Axis I disorders (n = 10)	Y	Y	Y	Y	Y	Y	Y
Discard study in Jackknife sensitivity analysis							
Bertsch *et al.*	Y	Y	Y	Y	N	Y	N
Brunner *et al.*	Y	Y	Y	Y	Y	Y	Y
Kuhlmann *et al.*	Y	Y	Y	Y	Y	Y	Y
Kuhlmann *et al.*	Y	Y	Y	N	Y	Y	Y
Minzenberg *et al.*	Y	Y	Y	Y	N	Y	N
Niedtfeld *et al.*	Y	Y	Y	Y	Y	Y	Y
O’Neill *et al.*	Y	Y	Y	Y	Y	Y	Y
Soloff *et al.*	Y	N	Y	Y	Y	Y	Y
Rossi *et al.*	Y	Y	Y	Y	Y	Y	Y
Rusch *et al.*	Y	Y	Y	Y	Y	Y	Y
Vollm *et al.*	Y	Y	Y	Y	Y	Y	Y

*Abbreviation*: SMA, supplementary motor area; MFG, middle frontal gyrus; IFG, inferior frontal gyrus; MTG, middle temporal gyrus; BA, Brodmann area; BPD, borderline personality disorder; L, left; R, right; N, no; Y, yes.

**Table 4:** Regional differences in gray matter volume between patients with drug-naïve BPD and healthy controls in subgroup meta-analysis of studies

Brain regions*	Maximum			Cluster	
	MNI coordinates x, y, z	SDM value	p value	Number of voxels	Breakdown
BPD > control					
R SMA & PCA	4,6,58	1.763	0.0001	1012	R SMA, BA 6; L SMA, BA 6;R PCA; B PMA
L precuneus	−4, −68, 36	1.289	0.0033	19	L precuneus, BA 7; L cuneus cortex
BPD < control					
R IFG	50,24,0	−2.316	0.00042	803	R IFG, opercular part, BA 48
					R IFG, triangular part, BA 45,47
					R insula, BA 47,48
L MTG	−60, −40, −2	−1.949	0.002	211	L MTG, BA21,20,22

*Abbreviation*: SMA, supplementary motor area; PCA, posterior cingulate cortex; PMC, primary moter corex;MFG, middle frontal gyrus; IFG, inferior frontal gyrus; MTG, middle temporal gyrus; BA, Brodmann area; BPD, borderline personality disorder; L, left; R, right; B, bilateral; SDM, seed-based *d* mapping; MNI, Montreal Neurological Institute.

^*^Regions identified by meta-analysis of coordinates from 7 data sets (voxel-wise p < 0.005 and voxel size > 10).
